# Supplementary material for: Extracellular Vesicle-Derived microRNA Crosstalk Between Equine Chondrocytes and Synoviocytes—An In Vitro Approach
Source: Int J Mol Sci. 2025 Apr 3;26(7):3353. doi: 10.3390/ijms26073353 (PMC11989968; doi:10.3390/ijms26073353)
Supplement: Supplementary file 1 [file ijms-26-03353-s001.zip › ijms-3509473-supplementary.pdf]

Supplementary material

Table S1. Demographics for control donor and recipient cells, stratified by cell type.

Characteristics	Donor cells		Recipient cells	
	**Chondrocytes (N=2)**	**Synoviocytes (N=2)**	**Chondrocytes (N=1)**	**Synoviocytes (N=1)**
Age, years				
Mean (SD)	7.5 (3.5)	7.5 (3.5)	10.0 (–)	10.0 (–)
95% CI	-24.3–39.3	-24.3–39.3	–	–
Min; Max	5; 10	5; 10	–	–
p value	>0.999[1]		–	
Sex, n (%)				
Female	1 (50.0)	2 (100.0)	0 (0.0)	1 (100.0)
Male	1 (50.0)	0 (0.0)	1 (100.0)	0(0.0)
p value	0.248[2]		–	
Joint macroscopic score (0–9)[3], n (%)				
0	0 (0.0)	0 (0.0)	0 (0.0)	0 (0.0)
1	0 (0.0)	1 (50.0)	0 (0.0)	0 (0.0)
2	2 (100.0)	1 (50.0)	1 (100.0)	1 (100.0)
≥3	0 (0.0)	0 (0.0)	0 (0.0)	0 (0.0)
Mean (SD)	2.0 (0.0)	1.5 (0.0)	2.0 (0.0)	2.0 (0.0)
95% CI	2.0–2.0	-4.9–7.9	–	–
Min; Max	2; 2	1; 2	–	–
p value	>0.999[4]		–	

[1] Calculated using a Mann-Whitney U test. [2] Calculated using a Chi-squared test. [3] Average of the scores obtained by three independent researchers using the Osteoarthritis Research Society International scoring system [16]. [4] Calculated using an unpaired t test.

CI, confidence interval; Max, maximum; Min, minimum; n, number of donors in a given category; N, number of donors included in the analysis set; SD, standard deviation.

Table S2. Top 10 diseases and functions associated with the predicted interaction network of uniquely expressed 5-EU labelled miRNAs from synoviocyte-derived EVs and their experimentally observed targets.

**Diseases & Functions**	**p-value**	**Number of molecules involved**[1]
Migration of tumor cell lines	4.36×10^{-34}	36
Proliferation of connective tissue cells	8.94×10^{-31}	28
Invasion of tumor cell lines	2.29×10^{-30}	33
Development of body trunk	2.69×10^{-30}	32
Invasion of cells	1.05×10^{-29}	34
Development of vasculature	9.28×10^{-29}	32
Maturation of cells	1.14×10^{-28}	24
Angiogenesis	1.63×10^{-28}	31
Growth of muscle tissue	1.79×10^{-28}	24
Growth of epithelial tissue	5.74×10^{-27}	27

[1] Number of molecules involved out of 44 (8 miRNAs uniquely expressed in synoviocyte-derived EVs and the corresponding 36 experimentally observed targets).

5-EU, 5-ethynyl uridine; EV, extracellular vesicle; miRNA, microRNA.

Table S3. Top 10 diseases and functions associated with the predicted interaction network of uniquely expressed 5-EU labelled miRNAs from chondrocyte-derived EVs and their experimentally observed targets

Diseases & Functions	p-value	Number of molecules involved[1]
Endometriosis	2.91×10^{-14}	9
Benign pelvic disease	3.52×10^{-13}	10
Pulmonary fibrosis or aplastic anemia	2.77×10^{-11}	8
Invasive cancer	1.56×10^{-10}	11
Epithelial-mesenchymal transition	1.67×10^{-10}	7
Vasculogenesis	2.88×10^{-10}	9
Metastatic gastrointestinal tract cancer	5.51×10^{-10}	7
Fibrosis of lung	6.03×10^{-10}	7
Advanced malignant gastrointestinal neoplasm	8.07×10^{-10}	7
Apoptosis of kidney cell	2.24×10^{-9}	6

[1] Number of molecules involved out of 12 (3 miRNAs uniquely expressed in chondrocyte-derived EVs and the corresponding 9 experimentally observed targets).

5-EU, 5-ethynyl uridine; EV, extracellular vesicle; miRNA, microRNA.

Table S4. Experimentally observed targets for the differentially expressed miRNAs between EV-recipient chondrocytes (RNA from EV-donor synoviocytes) and EV-recipient synoviocytes (RNA from EV-donor chondrocytes), filtered for cartilage, chondrocytes and osteoblasts.

**miRNA**	**Symbol**	**Gene name**
hsa-miR-143	IGFBP5	Insulin like growth factor binding protein 5
	BCL2	B-cell lymphoma 2 apoptosis regulator
hsa-miR-181a	BCL2	B-cell lymphoma 2 apoptosis regulator
	MMP14	Matrix metalloproteinase 14
	GRIA2	Glutamate ionotropic receptor AMPA type subunit 2
hsa-miR-199a-5p	SIRT1	Sirtuin 1
hsa-miR-21	RECK	Reversion-inducing cysteine-rich protein with Kazal motifs
	IL6R	Interleukin 6 receptor
	TNF	Tumor necrosis factor
hsa-miR-23b	CXCL12	C-X-C Motif Chemokine Ligand 12
	IL6R	Interleukin 6 receptor
	HES1	Hes family bHLH transcription factor 1
	SMAD4	Mothers against decapentaplegic homolog 4
	NOTCH1	Notch Receptor 1
hsa-miR-27b	PPARG	Peroxisome Proliferator Activated Receptor Gamma
	THRB	Thyroid Hormone Receptor Beta
	RXRA	Retinoid X Receptor Alpha
	FASN	Fatty acid synthase
	IGF1	Insulin-like Growth Factor 1
	NOTCH1	Notch Receptor 1
	MMP13	Matrix metalloproteinase 13
	SMAD4	Mothers against decapentaplegic homolog 4
hsa-miR-31	CASR	Calcium sensing receptor

EV, extracellular vesicles; miRNAs, microRNA.

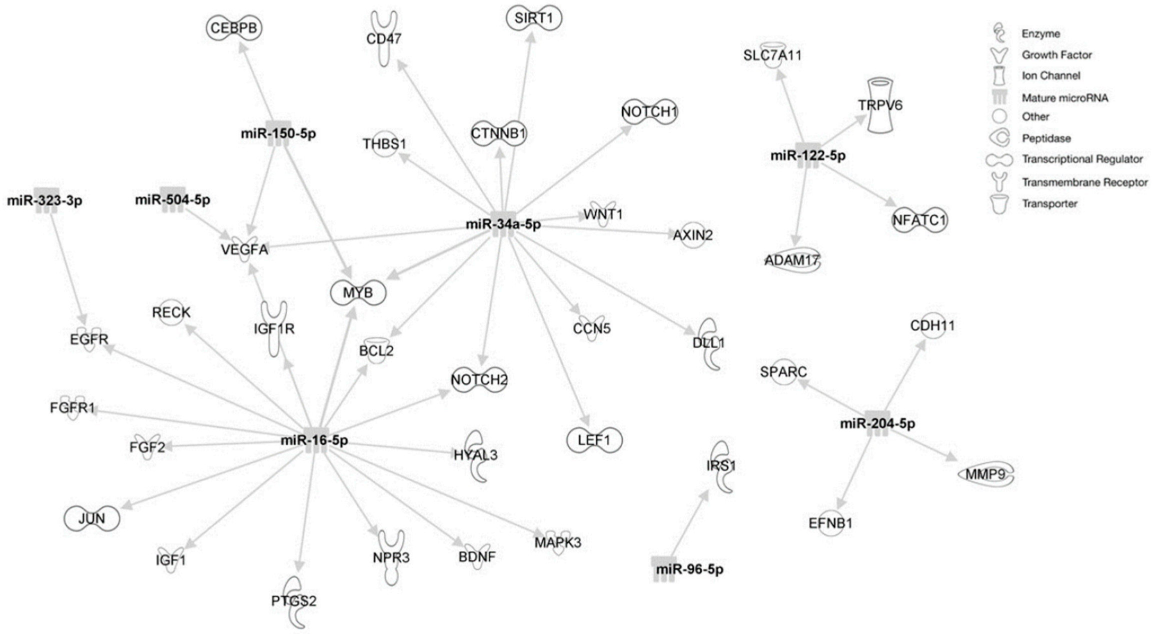

Figure S1. Interaction network of 5-EU labelled miRNAs uniquely identified in EV-recipient chondrocytes (RNA from EV-donor synoviocytes) and their experimentally observed targets in chondrocytes, osteoblasts and cartilage tissue. miRNAs are represented in gray and experimentally predicted targets are represented in white. Arrows represent direct interactions. ADAM17, A disintegrin and metalloprotease 17; AXIN2, axin 2; BCL2, B-cell lymphoma 2 apoptosis regulator; BDNF, brain-derived neurotrophic factor; CCN5, cellular communication network factor 5; CDH11, cadherin 11; CEBPB, CCAAT/enhancer-binding protein beta; CTNNB1, catenin beta 1; DLL1, delta like canonical Notch ligand 1; EFNB1, ephrin B1; EGFR, epidermal growth factor receptor; FGF2, fibroblast growth factor 2; FGFR1, fibroblast growth factor receptor 1; HYAL3, hyaluronidase 3; IGF1, insulin-like growth factor 1; IRS1, insulin receptor substrate 1; JUN, Jun proto-oncogene; AP-1 transcription factor subunit; LEF1, lymphoid enhancer binding factor 1; MAPK3, mitogen-activated protein kinase 3; MMP9, matrix metallopeptidase 9; MYB, MYB proto-oncogene transcription factor; NFATC1, nuclear factor of activated T cells 1; NOTCH1, Notch receptor 1; NOTCH2, Notch receptor 2; NPR3, natriuretic peptide receptor 3; PTGS2, prostaglandin-endoperoxide synthase 2; RECK, reversion inducing cysteine rich protein with Kazal motifs; SIRT1, sirtuin 1; SLC7A11, solute carrier family 7 member 11; SPARC, secreted protein acidic and cysteine rich; THBS1, thrombospondin 1; TRPV6, transient receptor potential cation channel subfamily V member 6; VEGFA, vascular endothelial growth factor A; WNT1, Wnt family member 1.

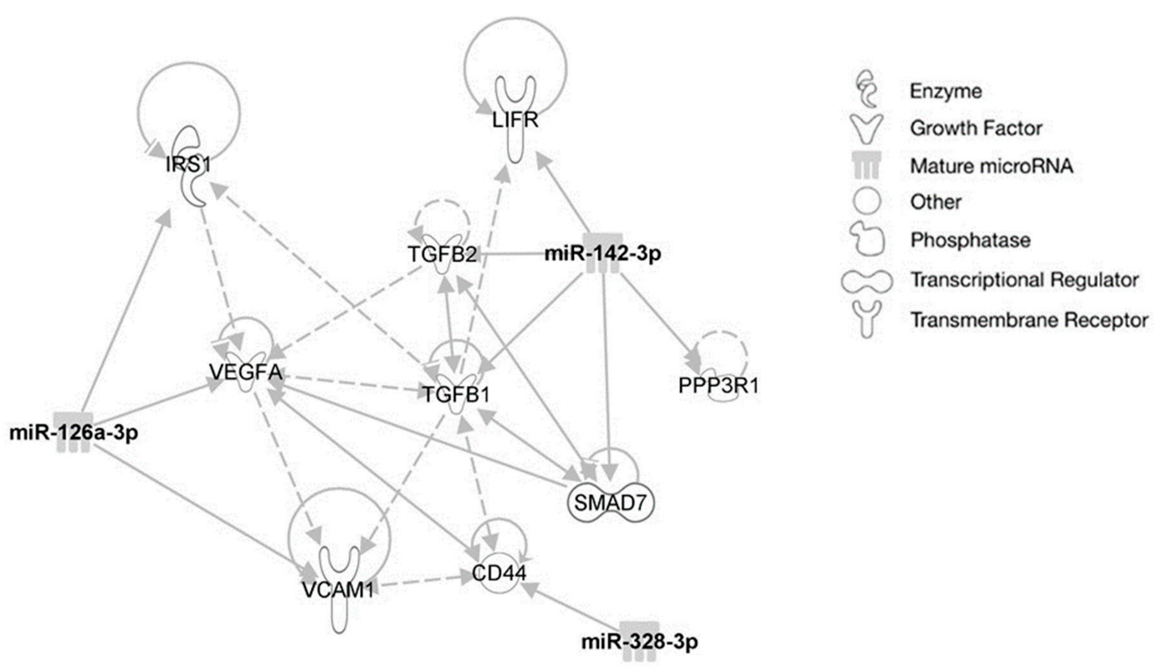

Figure S2. Interaction network of 5-EU labelled miRNAs uniquely identified in EV-recipient synoviocytes (RNA from EV-donor chondrocyte) and their experimentally observed targets in chondrocytes, osteoblasts and cartilage tissue. miRNAs are represented in gray and experimentally predicted targets are represented in white. Solid arrows represent direct interactions, and dashed arrows represent indirect interactions. IRS1, insulin receptor substrate 1; VCAM1, vascular cell adhesion molecule 1; VEGFA, vascular endothelial growth factor A; LIFR, leukemia inhibitory factor receptor; PPP3R1, protein phosphatase 3 regulatory subunit B alpha; SMAD7, mothers against decapentaplegic homolog 7; TGFB1, transforming growth factor beta 1; TGFB2, transforming growth factor beta 2.

Top Canonical Pathways

Name	p-value	Overlap
Role Of Osteoblasts In Rheumatoid Arthritis Signaling Pathway	2.30E-10	3.5 % 8/228
Osteoarthritis Pathway	2.64E-10	3.4 % 8/232
Tumor Microenvironment Pathway	1.63E-09	3.9 % 7/178
Hepatic Fibrosis / Hepatic Stellate Cell Activation	2.57E-09	3.7 % 7/190
Role Of Chondrocytes In Rheumatoid Arthritis Signaling Pathway	1.65E-08	4.3 % 6/140

Top Diseases and Bio Functions

Diseases and Disorders

Name	p-value range	# Molecules
Organismal Injury and Abnormalities	6.72E-05 - 4.60E-15	26
Inflammatory Disease	6.62E-05 - 2.31E-14	23
Cancer	6.62E-05 - 4.92E-14	26
Inflammatory Response	6.62E-05 - 5.48E-13	22
Gastrointestinal Disease	6.06E-05 - 2.30E-12	25

Molecular and Cellular Functions

Name	p-value range	# Molecules
Cellular Development	6.65E-05 - 1.05E-17	24
Cellular Growth and Proliferation	6.65E-05 - 1.05E-17	24
Gene Expression	4.13E-05 - 1.97E-16	15
Cell Death and Survival	6.72E-05 - 3.25E-14	22
Cellular Movement	6.80E-05 - 3.92E-14	22

Figure S3. Summary of IPA Core Analysis performed on differentially expressed miRNAs between chondrocytes and synoviocytes and filtered for experimentally observed relationships. Ingenuity Pathway Analysis; miRNA, microRNA.
